# Nutrition education and its relationship to body image and food intake in Asian young and adolescents: a systematic review

**DOI:** 10.3389/fnut.2024.1287237

**Published:** 2024-03-22

**Authors:** Baladandapla Shivappa Pushpa, Siti Norhedayah Abdul Latif, Sharimawati Sharbini, Zaidah Rizidah Murang, Siti Rohaiza Ahmad

**Affiliations:** ^1^PAPRSB Institute of Health Sciences, Universiti Brunei Darussalam, Gadong, Brunei; ^2^Sultan Hassanal Bolkiah Institute of Education, Universiti Brunei Darussalam, Bandar Seri Begawan, Brunei

**Keywords:** nutritional education, body image, Asian adolescents, perception, diet, food intake

## Abstract

**Background:**

The literature brings to light the unhealthy nutritional habits prevalent among Asian adolescents and their high level of body image dissatisfaction. This study aims to conduct a systematic review of the literature on the effect of nutritional education interventions on their nutritional knowledge and food intake behavior, attitude, practice, and body image.

**Methods:**

We searched relevant published studies in PubMed, Web of Science, Scopus, Science Direct, and Springer using the PICO framework and performed a quality assessment using the 10-point checklist adapted from the National Institutes for Health tool.

**Results:**

The majority of the nutritional education interventions improve unhealthy food intake and body image misperception, particularly on nutritional knowledge/self-efficacy, healthy dietary habits, physical activities, and fruit and vegetable intake. We also found a negative association with excess weight gain, obesity, and unethical weight reduction practices, leading to dissatisfaction with body image.

**Conclusion:**

These interventions can help address dietary problems and body image perception and support the development of future interventions.

## 1 Introduction

Obesity and excess weight gain are increasingly common issues among adolescents. Unhealthy diet choices and reduced physical activity are also prevalent and lead to a persistent positive energy balance (1). This, in turn, leads to excess weight gain among children and adolescents (2). Adolescents are particularly affected because they have higher nutritional needs as they undergo rapid growth and development. Significant physical, psychological, and social changes occur throughout this formative period of life. However, insufficient intake of essential nutrients such as carbohydrates, proteins, fats, vitamins, and minerals may harm their immediate growth and long-term development. Yet, a troubling pattern emerges where many young individuals, instead of prioritizing nutritious foods, opt for calorie-dense, nutrient-poor foods to meet their food cravings and stave their hunger. According to multiple reports, adolescents are more likely to consume unhealthy foods such as refined carbohydrates (snacks and beverages with added sugar), caffeine, and junk food than they are to consume fruit, milk, and vegetables (3–5). The following evidence highlights this: Most adolescents skip meals frequently, only up to 50% consume fruits and vegetables and use dietary supplements, 13.9% of the total energy intake of Korean adolescents comes from free sugar, significantly higher than the 5% recommended by the World Health Organization (WHO) (6), 53.9% of the beverages consumed contain added sugar, accounting for 20.5% of the daily sugar intake and 12.5% of high school pupils use more caffeine than is advised (7). This preference is what leads to an imbalance in their diet, a situation that can only be rectified through decisive efforts guiding the formulation of effective strategies promoting healthier eating habits.

Moreover, body image misconceptions distort the perception of one’s body and set unrealistic expectations of how one should look. Social media and advertising significantly perpetuate these misconceptions by showing idealized and often unattainable body types. It was reported that 24.2% of adolescents in middle and high school had negative body image issues despite having a normal body mass index (BMI) (8). This negative self-impression of body image may result in severe physical and mental effects (9) and may lead to melancholy, low self-esteem, binge eating, eating disorders, consuming unhealthy foods, and excessive weight loss practices, and this is a growing concern around the world (10, 11). It has been identified as a global problem that transcends geographical boundaries and affects individuals of all income levels and linguistic backgrounds (12, 13). Since proper nutrition is vital in promoting adolescents’ physical and mental health, a multifaceted approach that includes nutritional education and support for individuals struggling with body image is required. It is crucial to encourage a healthy relationship with food, promote self-acceptance, and focus on overall well-being rather than external appearance. Negative attitudes toward food and a distorted body image not only contribute to obesity but also exert a profound influence on an individual’s mental and physical well-being (14). Developing healthy eating habits during adolescence could prevent obesity, type 2 diabetes, cardiovascular disease, and other disorders. They also promote mental alertness and increased cognitive function and catalyze positive thoughts about one’s appearance and self-worth. Nutritional interventions aimed at fighting obesity can also be used to decrease the incidence of excessive food intake and wrong body image assessment.

Nutritional education interventions aim to improve nutrition-related knowledge, attitudes and practices. This provides individuals with skills and information to help them improve their diet, lifestyle, behavior and emotional well-being. Some of the common methods to deliver nutrition education interventions include lectures and counseling. Increasingly, web resources, social media, video-based learning, mobile apps and game-based learning are also being used. In an educational setting, nutritional interventions are integrated into the classroom through nutrition programs, peer education and healthy school meal initiatives. The nutrition curriculum often includes concepts of food production and preparation, healthy eating by making the right food choices, lifestyle modification, and cultural aspects of nutrition. Hands-on assignments include cooking classes, kitchen safety and field trips. Notably, school meal programs typically include the promotion of locally and regionally produced foods or food products, which contributes to a comprehensive approach. The success of these interventional measures depends on the active involvement of parents, the community and teachers, with a focus on a holistic approach.

The literature highlights the unhealthy nutritional habits prevalent among Asian adolescents and multiple cross-sectional studies have identified this subpopulation as exhibiting the highest level of dissatisfaction with their body image. Improving nutritional education interventions would lower the incidence of obesity among them (5). We can further note that many educational interventions have succeeded in addressing unhealthy eating behavior and body image dissatisfaction in a large number of countries. However, to date, there are no studies that systematically review the efficacy of these interventions. This study fills this gap and thoroughly analyzes the impact of various nutritional education interventions on nutritional knowledge and related food intake behavior, attitude and practice, and body image in the Asian young and adolescent population.

## 2 Materials and methods

We aligned the systematic review methodology with our research goal of exploring the effect of nutritional educational intervention on nutritional knowledge, attitude, practice, and body image in the Asian young and adolescent population. This procedure followed the six-stage technique of a systematic literature review, beginning with the creation of protocols and continuing into eligibility criteria, search strategy, study evaluation, data extraction, and result synthesis.

### 2.1 Development

The review protocol explains the review’s historical context, the publications chosen for review, and how it tackles publication quality evaluation and data retrieval. We employed the Preferred Reporting Items for Systematic Reviews and Meta-Analyses (PRISMA) statements as recommended for conducting a systematic literature review (15).

### 2.2 Research question

We used the problem/population, intervention, comparison, and outcome (PICO) framework to define the review questions. This framework informs the search string and the search strategy. Table 1 shows the list of terms comprising each of the four components of the PICO framework we have used to frame the two research questions. The review aims to answer two research questions: Research question 1 (RQ1): How does nutritional education intervention affect food intake behavior and its related outcomes? Research question 1 (RQ2): How does nutritional education intervention affect body image perception?

**Table 1:** PICO framework for outlining the research questions.

Problem/population	Intervention	Comparative intervention	Outcome
Problem: Unhealthy food intake and body image dissatisfaction. Population: Asian adolescents, young adults, university, college or high school students	Nutritional education, dietary intervention, healthy eating intervention, body image intervention	Nutritional education vs., no intervention	Healthy food intake and positive perception of body image, weight reduction, nutritional knowledge/education, increased physical activities

### 2.3 Search strategy

We selected and then searched five electronic databases PubMed, Web of Science, Scopus, Science Direct, and Springer. The search was conducted on the 17th of March, 2023. Search terms included terms such as ‘nutritional knowledge,’ ‘dietary intervention,’ ‘nutritional knowledge,’ ‘nutritional attitude,’ ‘eating practice,’ ‘body image perception,’ and ‘body image satisfaction’ and different Asian geographical locations. Search terms were combined using Boolean operators “AND” and “OR” in the title and abstract index. The search was limited to articles published between January 2000 and December 2022.

### 2.4 Eligibility criteria

We defined the criteria for including or excluding retrieved studies. Table 2 shows the inclusion and exclusion criteria. No studies were excluded based on the year of publication.

**Table 2:** Criteria for inclusion and exclusion.

Inclusion criteria	Exclusion criteria
English language	Non-English language
Empirical studies	Secondary studies
Original full-text articles	Conference abstracts, commentaries, dissertations, notes, letters, review papers, gray literature, case reports
Nutritional education intervention toward study outcome	Other intervention
Asian adolescents aged 9–25 y/o	Other regions, children aged <9 y/o and >25 y/o
Directly answers research question	Not in line with research question
Human model	Animal model
Reports food intake and body image perception	

### 2.5 Article screening

We employed Covidence screening software to manage and screen the bibliographic data from the five electronic sources. The software automatically excluded duplicate articles. The references were upvoted on the software using the inclusion and exclusion criteria. We examined both article’s title and abstract to determine their alignment with the research objectives. Then we read the full text of the shortlisted papers. Two independent reviewers screened the article’s titles, abstracts, and full text to reduce bias. All eligible articles were thoroughly reviewed, and the discrepancy between the reviewers during the screening was resolved by discussion and consensus.

### 2.6 Study appraisal

The objective of this appraisal was to evaluate the quality of the articles in terms of their methodology and to address the possibility of bias in their planning, execution, and analysis. Following the inclusion criteria, two critical reviewers conducted a rigorous evaluation of all articles selected for this systematic review. The quality assessment was performed using the 10-point checklist adapted from the National Institutes for Health tool (16) for appraising observational, cohort, and cross-sectional studies. Table 3 shows the 10 items on the checklist considered in this study which help assess the quality of the articles. The 10 items were evaluated using the dichotomous *Yes or No* rating method. The quality of evaluated studies was subcategorized as high (above 70% of *Yes* responses), moderately high (50–69% of *Yes* responses), or low (below 50% of *Yes* responses), depending on their assessment score.

**Table 3:** Assessment checklist items (adapted from the National Institutes for Health).

Q1	Was there an adequate definition of the research question/objective?
Q2	Was there an adequate definition of the study population?
Q3	Was there an indication of a ≥50% participation rate?
Q4	Were there inclusion and exclusion criteria for all participants?
Q5	Was there justification for sample size/description of power and effect sizes?
Q6	Was there an adequate definition of exposure measures?
Q7	Was there an adequate definition of outcome measures?
Q8	Was there an assessment of confounding variables’ effects?
Q9	Was statistical analysis carried out for all groups?
Q10	Was there adequate reporting with information regarding statistical methods?

### 2.7 Data extraction

Data was extracted from the studies using an electronic data extraction form developed with Microsoft Excel. The characteristics of each study were summarized under various headings, allowing the most important findings to be synthesized. The information extracted includes the following: (i) primary author’s information and affiliations, (ii) publication year, (iii) country, (iv) aim of the article, (v) study design, (vi) sample characteristics including the educational level of the participants, sample size, age and gender, (vii) description of the nutritional interventions used, (viii) duration of the experiment to follow-up period, (ix) method of assessing the intervention outcome, (x) outcome, (xi) statistical analysis, (xii) results, and (xiii) the main findings of the articles. After data extraction, we organized and summarized the article characteristics to answer the systematic review questions.

## 3 Results

This section presents the selection process of the papers in the systematic review and the results of both the quality assessment and data extraction.

### 3.1 Study selection

**Figure 1:**
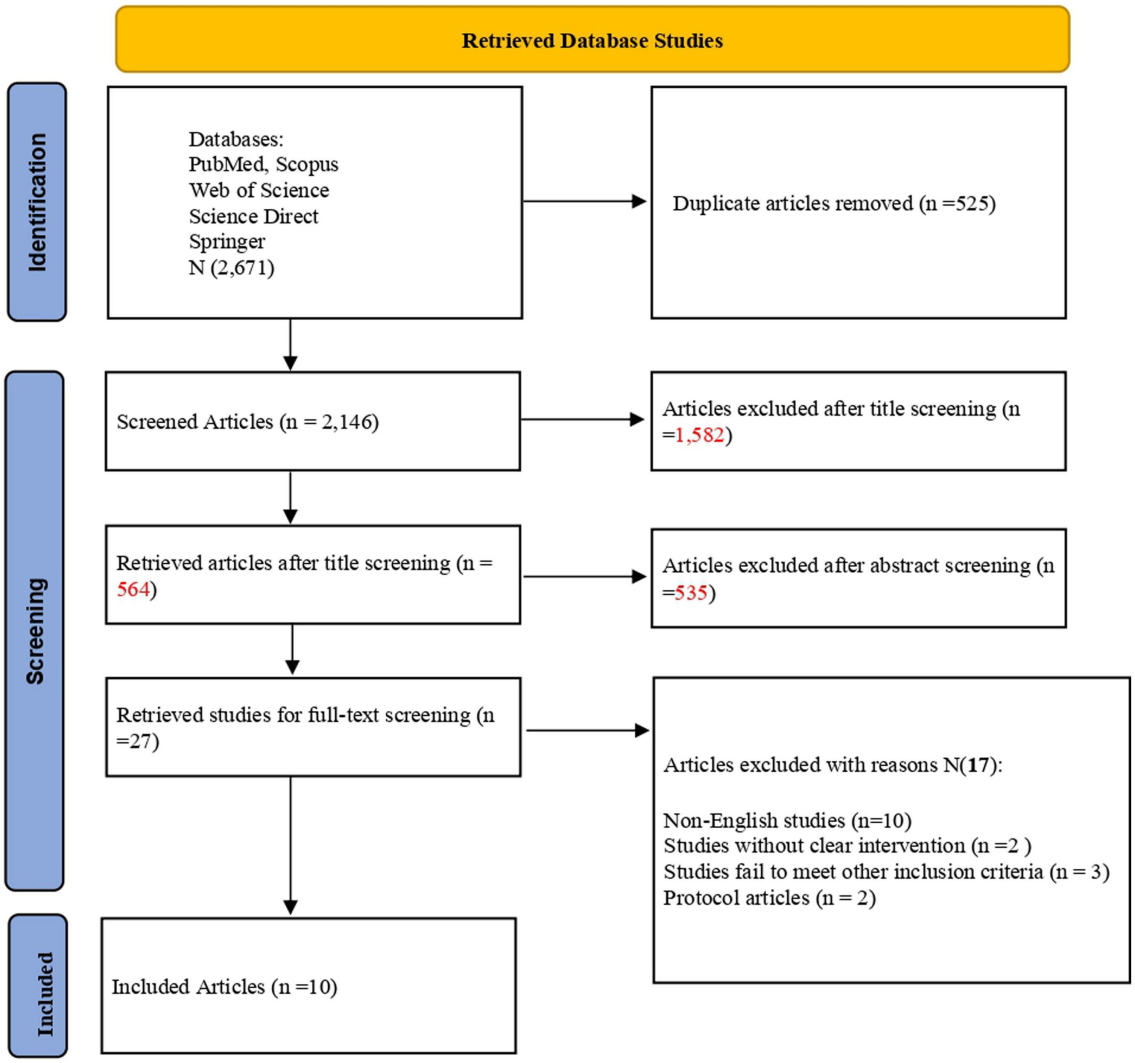
PRISMA diagram showing screening process.

We retrieved 2,671 articles from the five electronic databases and exported them to the Covidence software for reference management. The software excluded 525 duplicate records. Screening the titles and abstracts of the remaining studies disqualified 2,119 articles as they did not align with the research question. Two independent reviewers evaluated the remaining 27 studies and based on the inclusion criteria excluded 17 studies. Finally, 10 articles met the inclusion criteria and were included in the review. Figure 1 displays the PRISMA chart that explains the screening procedure.

### 3.2 Quality assessment

We evaluated the 10 articles’ methodological and reporting quality. The detailed results are presented in Table 4 as outlined in Section 2.6. All of them obtained a score of more than 70% and thus were of good quality. Only 10% of the articles —one article— met all 10 items on the checklist and therefore scored 100%. Eight studies scored between 80 and 90%, while the remaining two studies scored 70%.

**Table 4:** Quality assessment of the studies.

Study	Q1	Q2	Q3	Q4	Q5	Q6	Q7	Q8	Q9	Q10	Yes (%)	Quality
Marliya and Muhammad (17)	Yes	Yes	Yes	Yes	No	Yes	Yes	No	Yes	Yes	80	High
Shahril et al. (18)	Yes	Yes	Yes	Yes	No	Yes	Yes	Yes	Yes	Yes	90	High
Wang et al. (19)	Yes	Yes	Yes	Yes	No	Yes	Yes	No	Yes	Yes	80	High
Singhal et al. (20)	Yes	Yes	Yes	Yes	No	Yes	Yes	No	Yes	Yes	80	High
Chen et al. (21)	Yes	Yes	Yes	Yes	No	Yes	Yes	No	Yes	Yes	80	High
Lee et al. (22)	Yes	Yes	Yes	Yes	No	Yes	Yes	Yes	Yes	Yes	90	High
Koo et al. (23)	Yes	Yes	Yes	Yes	No	Yes	Yes	Yes	Yes	Yes	90	High
Sharif Ishak et al. (24)	Yes	Yes	Yes	Yes	Yes	Yes	Yes	Yes	Yes	Yes	100	High
Tse and Yuen (25)	Yes	Yes	Yes	No	No	Yes	Yes	No	Yes	Yes	70	High
Yeh et al. (26)	Yes	Yes	Yes	Yes	Yes	Yes	Yes	No	Yes	Yes	90	High

Q1–Q10 represent the checklist items highlighted in Table 3.

Specifically, we can note that all the studies had a defined research question and an adequate description of the study population, at least 50% of the study population had participated, both the exposure and outcome measures were adequately defined, statistical analysis had been conducted and they all had adequate reporting. As listed in Table 4, all the studies outlined inclusion and exclusion criteria for all participants (17–24, 26) except for Tse and Yuen (25). Conversely, only Sharifa Ishak et al. (24) and Yeh et al. (26) justified the sample size and described power and effect sizes. Finally, only four studies (18, 22–24) included an assessment of confounding variable effects.

### 3.3 Result synthesis

The selected studies covered five Asian countries. Most of the articles examined Malaysian (18, 23, 24) and Chinese adolescents (19, 25, 26), whereas others focused on Korean adolescents (21, 22), Indonesian adolescents (17), and Indian adolescents (20). The studies were published between 2009 and 2021. The study population of the reviewed articles included university undergraduates, middle and high school students, secondary school students, and 4th, 5th, 6th, 8th, and 11th-grade students. All the participants were aged 9–25 years old. Male students who participated in the experiments were slightly older than the female participants.

These studies adopted various study designs like randomized controlled trials (RCT) (17, 21), non-randomized controlled trials (19), cross-sectional (20, 22) quasi-experimental design (23, 24, 26), pre-post study design (25), and cluster RCT (18). Of the 61,893 Asian adolescents enrolled in the different studies, 29,670 participants received nutrition education intervention, whereas 32,223 did not.

The nutritional education interventions covered in the articles were the following: animation movies and lectures (17); a multimodal nutrition education module (18, 20); a WeChat app (19); iStartSmart for teens educational program using an online format consisting of a short video (21) and an e-book (22); GReat-Child Trial™ based on social cognitive theory improved knowledge, attitudes and practices (23); module delivery focused on healthy eating, positive body image, an active lifestyle (24); tailor-made nutritional education (25); and an educational program on body image (26). The duration of the experimental follow-up period varied across the studies, ranging from 3 to 36 weeks after the administration of the intervention.

The outcomes measured in the articles were the following: obesity and weight gain (17), dietary intake habits (18, 25), physical fitness tests (19), anthropometry (19–21, 24), nutritional change and blood pressure (21), sedentary activity (21), self-efficacy and quality of life (21), body image misperception (22), breakfast frequency (22), knowledge-attitude-practice towards whole grain consumption (23), level of physical activity (19, 21, 25) and perceptional, attitudinal, and behavioral aspects of body image (26).

The statistical analyses used by the authors to measure the significance of the outcomes included independent samples *t*-test (17, 20, 22), paired samples *t*-test (17, 20, 25), Wilcoxon signed rank test (17, 19), spearman correlation (17), analysis of covariance (ANCOVA) and adjusted effect size (18, 23, 24), McNemar test (20), linear mixed effects models and regress models (21), weighted chi-square test (22, 24), and weighted multivariate logistic regression analysis (22). One included study did not report statistical methods (26).

### 3.4 Intervention outcome

The interventions reviewed positively affected dietary intake behaviors and were especially effective in reducing the consumption of unhealthy food, thus leading to a reduction in body image misperception. Table 5 lists the 31 different outcomes covered by the 10 articles reviewed by source and a detailed synthesis of results is summarized in Table 6.

**Table 5:** Impact of nutritional education intervention.

S/n	Effects of nutritional education interventions	References
1	Improve nutritional knowledge/self-efficacy	(17, 18, 20, 21, 23–25)
2	Increase healthy dietary intake	(18–20, 22, 25)
3	Improve physical activities	(19–22, 25)
4	Increase vegetable consumption	(17, 19, 22)
5	Increase fruits intake	(18, 22)
6	Reduce dietary intake	(17)
7	Reduce energy intake	(17)
8	Reduce fried food consumption	(17, 25)
9	Reduce consumption of snacks	(17, 25)
10	Reduce consumption of sugar	(17)
11	Decrease processed and deep-fried food intake	(18, 25)
12	Increase energy intake with fish and egg	(18)
13	Increase carbohydrate intake	(18)
14	Increase minerals and vitamins intake	(18)
15	Increase milk and dairy product intake	(18, 20)
16	Decrease consumption of aerated drinks	(20, 25)
17	Decrease energy-dense unhealthy foods	(20, 25)
18	Increase the proportion of tiffin and fruits in tiffin (packed lunch) brought to school.	(20)
19	Decrease in sagittal abdominal diameter	(20)
20	Reduction of sugary beverage intake	(20–22)
21	Reduction of TV and computer time	(21)
22	Decrease fast food consumption	(21, 22, 25)
23	Reduce breakfast skipping	(22)
24	Increase knowledge, attitude, and practice of whole grain consumption	(23)
25	Decrease in mean waist circumference	(20)
26	Decreased waist-to-hip ratio	(20)
27	Decrease fasting blood glucose, triglycerides	(20)
28	Reduction in BMI	(21)
29	Lower body image misperception	(22)
30	Decrease in abdominal obesity	(24)
31	Increase body image satisfaction (attitudinal, perceptional and behavioral)	(26)

**Table 6:** Synthesis table.

Authors	Year	Country	Aim	Design	Educational level	Sample size	Age	Gender	Intervention	Duration	Method of assessment	Assessed outcome	Statistical test	Outcome	Main findings
Marliya and Muhammad (17)	2019	Indonesia	Impact of educational movie on nutritional knowledge, eating habit and dietary intake	Controlled intervention design	High school	N80 (I:40; C:40)	15–17 years	NR	Control: Nutritional lecture only; Intervention: Lecture + a short educational movie on nutrition	4 weeks	BMI calculator, Nutrition knowledge questionnaire, SQ-FFQ questionnaire,	Obesity/overweight: Nutritional knowledge, eating habit and dietary intake	Independent samples *t*-test, Paired samples *t*-test, Wilcoxon signed rank test, spearman correlation	Nutritional knowledge: Both groups had increment in nutritional knowledge after the intervention but movie group had higher increment than those in control group (*p* < 0,05). Dietary intake: reduced in both groups. Subjects in movie group had significantly higher reduction in energy intake after the first month of intervention (*p* < 0,05). Eating habits: subjects in movie group significantly reduced consumption on fried foods (*p* < 0,0001), snacks (*p* = 0,037), sugar (*p* = 0,015) and increased consumption on vegetables (*p* = 0,029)	The study showed that implementing nutritional interventions, such as educational films on a school-based promotion of dietary guidelines, significantly increased adolescents’ nutritional knowledge, improved their eating habits, and significantly decreased their energy intake, which are moderators of overweight/obesity.
Shahril et al. (18)	2013	Malaysia	Impact of 10-week multimodal nutrition education intervention on dietary intake	Cluster randomized Controlled Tria	University undergraduates	N417 (I:178; C:202)	*M* = 19.2 ± 1.1	NR	Control: No treatment received; Nutritional education intervention [NEI] via conventional lecture, brochures, and text messages.	10-week	Dietary questionnaires	Dietary intake	Analysis of Covariance (ANCOVA) and adjusted effect size	Dietary intake: Participants that receive intervention were seen to significantly improved their dietary intake by increasing their energy intake, carbohydrate, calcium, vitamin C and thiamine, fruits and 100% fruit juice, fish, egg, milk, and dairy products as opposed to controlled participants. Also, those that receive intervention significantly decreased their processed food intake	The results of the study showed that a multimodal nutritional education intervention (NEI) with an emphasis on promoting healthy eating is a successful strategy for raising dietary intakes among university students.
Wang et al. (19)	2019	China	Impact of social media-based intervention on promoting healthy lifestyle	Non randomized control intervention design	University undergraduates	N110 (I: 87; C: 23)	Median: 18 (1.0)	F:65 M:45	Control: No reminder Intervention: social media-based health promotion program (dietary advice, exercise encouragement, healthy habits reminders on WeChat)	3 weeks	Questionnaires on demographic details, dietary intake estimates and physical activity information	Eating habits, physical fitness tests and anthropometry	Wilcoxon signed rank test	Participant enrolled to intervention showed significant improvement in healthy food intake for all food kinds (*p* < 0.05), and an improvement in physical activities [PA] level (*p* = 0.004) over 21 days.	The study indicated that social media-based initiatives to promote physical activity and healthy eating can significantly enhance this behavior among undergraduate students and that these initiatives are highly feasible for wider adoption.
Singhal et al. (20)	2010	India	Effects of controlled school-based multi-component model of nutrition and lifestyle interventions on behavior modification, anthropometry and metabolic risk profile	Cross-sectional	11th grade	N201 (I: 99; C: 102)	(15–17 years)	F:80; M:121	Control: No intervention received. Intervention: Nutrition and lifestyle intervention based on the Dietary Guidelines for Indians (delivered through lectures and focus group discussions)	24 weeks	Anthropometric measurements and	Change nutrition-related knowledge, attitude, lifestyle practices, food frequency and body image	Student’s paired *t*-test, independent samples *t*-test, McNemar test	Significant improvement in nutritional-related knowledge among participant enrolled to intervention. Significant decrease in consumption of aerated drinks (15.1%; *p* < 0.001) and energy-dense unhealthy foods (8.9%; *p* = 0.03), and significant higher proportion of tiffin (packed lunch) (14.9%; *p* = 0.004) and fruit in tiffin (30.7%; *p* < 0.001) brought to school were observed in intervention group as opposed to control. Finally, Significant decrease in mean waist circumference [*p* = 0.02, 95% confidence interval (CI) = −2.43, −0.17], sagittal abdominal diameter (*p* < 0.001, 95% CI = -0.82, −0.09), waist-to-hip ratio (*p* = 0.02, 95% CI = -0.03, −0.004), triglyceride (95% CI: −54.20 to −24.04, *p*;0.001) and fasting blood glucose (*p* = 0.05, 95% CI = -0.09, 5.00)	The results showed that a multi-component approach of nutrition and lifestyle education can successfully increase nutrition-related knowledge, eating behaviors, and lifestyle practices, leading to positive improvements in Asian Indian adolescence anthropometric and biochemical profiles.
Chen et al. (21)	2018	South Korea	Short-term efficacy of a smartphone-based intervention for Chinese American adolescents who are overweight or obese and factors associated with decreased body mass index (BMI)	Randomized Controlled Trial	NR	N40 (I: 23; C: 17)	13–18 years	F: 17 (42.5%); M: 23 (57.5%)	Control: general health information. Intervention: Culturally educational program for weight management	12 weeks	Anthropometry survey, food consumption survey, sedentary activity survey, physical activity self-efficacy and healthy eating self-efficacy survey, Pediatric quality of life survey.	Anthropometrics, blood pressure, levels of physical and sedentary activity, diet, self-efficacy, and quality of life	Linear mixed-effects models and regression models	Intervention group had significant reduction in BMI (*z* = −4.89, *p* < 0.001), sugary beverage (*z* = −0.44, *p* = 0.001), and TV and computer time (*z* = −0.51, *p* < 0.001) and increase nutrition self-efficacy, and physical activity. Also, decrease fast food consumption and increase physical activities reduce BMI. Female participant + decrease sugary food result in reduce BMI	There is a lot of potential for reducing obesity and boosting adherence to a healthy lifestyle using a smartphone-based intervention that is culturally relevant. Adolescents who are overweight or obese can lower their BMI by cutting back on sugary drinks, fast food, and sedentary activities.
Lee et al. (22)	2021	Republic of Korea	Association between nutrition education, dietary habits, and body image misperception in adolescents	Cross-sectional design	Middle and high schools	N60,389 (I:28,863; C:31,526)	12–18 years	F:50%; M:50%	Control: uneducated (non-EDU) Intervention: Nutrition-educated (EDU)	24 months	Online questionnaires	Body image misperception, breakfast frequency, dietary behaviors	Weighted independent *t*-test, weighted chi-square tests, weighted multi-variate logistic regression analysis	Intervention group had desirable dietary behaviors (fruits, milk, and vegetables intake) more than control group. Also, less associated with skipping breakfast. Lower body image misperception in the intervention compared to control.	Nutrition education have positive effects on healthy dietary behaviors. It also shows a negative association with body image misperception, confirming the importance of nutrition education at school.
Koo et al. (23)	2019	Malaysia	Evaluation of the feasibility and acceptability of the GReat-Child Trial™, as well as to determine the changes in knowledge, attitudes and practices (KAP) of whole grains consumption among overweight/obese children	Quasi-experimental design	4th and 5th grade	N63 (I:31; C:32)	9–11 years	F:30; M:33	Control: No treatment received; Intervention: multi-component whole grain intervention (GReat-Child Trial)	36 months	Knowledge, attitudes and practices (KAP) questionnaires	KAP towards whole grains consumption	ANCOVA	Significant increase in KAP scores towards whole grains consumption *p* < 0.001. Outcome at the 9th month were more significant than the first month	These findings indicate that this intervention made a positive impact on improving children’s KAP on whole grains. We anticipate the GReat-Child Trial™ to be a program that could be incorporated into school interventions to improve whole grain consumption among Malaysian children for obesity prevention
Sharif Ishak et al. (24)	2020	Malaysia	Evaluating the effectiveness of the EPaL program on knowledge, attitudes and practices on healthy lifestyle and body composition among adolescents	Quasi experimental design	Secondary school	N76 (I:34; C:42)	13–14 years	F:38; M:38	Control: no intervention received; Intervention: ‘Eat Right, Be Positive About Your Body and Live Actively’ (EPaL)	16 weeks	Anthropometric measurements; knowledge, attitude, and practice survey	Anthropometric measures, knowledge, attitude, and practice	ANCOVA and Chi-square	Significant increase in knowledge score in intervention group. Attitude and practice were not significantly different in the two group. Also, the proportion of participants who had abdominal obesity in the IG decreased from 17.6% at Pre to 14.7% at Post II, although not significantly different from 16.7% at Pre to 21.4% at Post II reported in the control group.	Despite no significant reduction of body composition, this program shows the positive effect on the adolescents’ knowledge regarding healthy lifestyle
Tse and Yuen (25)	2009	China	Effects of providing a nutrition education program for teenagers: dietary and physical activity patterns	Experimental pre-post study design	Year 8 secondary school	N203	13.5 years.	F: 88; M:115	Control: no intervention, Intervention: Education program on dietary intake and body image	12 months	Physical activity survey, dietary/food intake survey	Physical activity, dietary/food intake	Chi-square, paired samples *t*-test	There was a marked gain in knowledge upon completion of intervention. At three-month follow-up, positive outcome on physical and dietary intake level	The educational initiatives in dietary habits and physical activities proved to be effective in encouraging the teenagers to eat more healthily and to adopt an active lifestyle
Yeh et al. (26)	2011	China	Development and effectiveness of a school program on improving body image among elementary school students in Taiwan	Quasi experimental design	5th and 6th grade	N314 (I:112, C: 202)	10.72 years	F:53.6; M:46.4	Control: no intervention, Intervention: Nutrition education program	8 week	Body image survey, Diet and vomiting behaviors	Perceptional (estimation of body size), attitudinal (body satisfaction) and behavioral (diet and vomiting to lose weight) aspects of body image	NR	Intervention students showed a greater increase in body satisfaction. No statistically significant differences in the perceptional and behavioral aspects of body image	This study demonstrated that a school program improved body satisfaction among elementary school students

#### 3.4.1 Nutritional education intervention and dietary knowledge

Five studies emphasized a significant improvement in dietary/nutritional knowledge among adolescents (17, 20, 21, 24, 25). These studies revealed high levels of knowledge across different areas of diet and nutrition after intervention. The participants demonstrated increased nutritional knowledge of dietary types, attitudes and behavior and physical activities, as well as a new awareness of body image following the intervention. In particular, their new knowledge included information about simple and complex carbohydrates, the concept of empty calories, sources, adverse effects of trans-fat, high-fat dairy products, dietary fiber, refined grains, whole grains, sugary contents, and glucose elevators.

#### 3.4.2 Nutritional education intervention and dietary attitude and practice

All of the articles found interventions to have a significant effect on the nutritional attitudes and practices of Asian adolescents (17–26). These attitudes and practices revolve around healthy food intake behaviors such as increasing the consumption of fruits and vegetables (17–20, 22, 23) and the proportion of fruits in packed lunches brought to school (20), increasing the consumption of healthy carbohydrates, minerals and vitamins, whole grain, and dairy products (18, 20, 23, 25), and reducing the consumption of unhealthy foods (energy-dense, fried, sugary, processed, junk foods, snacks, carbonated drinks and excessive food intake) (17, 18, 20, 21, 25).

There was also a lower exposure to TV and screens, a lower intake of food in canteens, a lower consumption of fast food and a reduction in the number of participants skipping breakfast (20, 21, 23). One study reported that 93% of participants felt happy with the lifestyle changes made after the intervention (21), while another study reported a significant change in knowledge, attitudes, and practice (23). Conversely, one study reported an increase in knowledge but no change in practices and attitudes (24). Another study highlighted that more participants recognized that eating a serving of unhealthy foods would harm their health and thus decrease their intake of food from restaurants, kiosks, and eateries (20).

#### 3.4.3 Nutritional education intervention and physical activity

Five studies identified that nutritional intervention significantly improved adolescent physical activity levels (19–22, 25). Reported physical activity included increasing the duration of quiet squats (19) and plank time (19), as well as incorporating 30–60 min of physical activity per day or participating in physical activity more than 4 days per week (20). These adolescents demonstrated less sedentary lifestyles, more hours of exercise (25), and more participation in household chores (25). There was also a significant increase in the number of adolescents who walked instead of taking transport as an activity (25).

#### 3.4.4 Nutritional education intervention and body weight

Four studies highlighted the significant effect of a nutritional education intervention on adolescents’ body weight and its associated factors (20, 21, 23, 24). According to these studies, nutritional education interventions significantly reduced sagittal abdominal diameter (20), abdominal obesity (24), body mass index (21), fasting blood glucose level (20), waist circumference, and waist-to-hip ratio, which significantly altered anthropometry, body composition, and metabolic parameters (20). In another study, more participants correctly identified their body size at pre-intervention, post-intervention, and 3 months after the intervention (26).

#### 3.4.5 Nutritional education intervention and body image

Several studies demonstrated lower body image misperception (23) and a significant increase in body image satisfaction 3 months after intervention (26). Surprisingly, no significant difference in the scores for vomiting behavior post-food intake was reported (26). An article reported slightly higher overall satisfaction with physical appearance and fewer participants who avoided clothes that made them look overweight (20).

## 4 Discussion

The systematic literature review analyzed the findings of 10 articles retrieved from five electronic databases—PubMed, Web of Science, Scopus, Science Direct, and Springer—which focus on the impact of nutritional education interventions on nutritional knowledge and related food intake behavior, attitude, practice, and body image among the Asian young and adolescent population. The PICO framework was used to formulate the review questions, ensuring a focused and systematic research strategy. All the articles met the inclusion criteria and were checked for methodological quality using the National Institutes for Health tool. The guidelines of the PRISMA and Cochrane were thoroughly followed.

The studies cover very different interventions and outcomes, but because of this diversity and the high degree of overlap between them, we can infer meaningful conclusions. We explored multiple interventions of nutrition education, including lectures, movies, social media, text messages and notes (17–19). These interventions impacted key factors such as nutritional knowledge/self-efficacy, healthy dietary habits, physical activity, and fruit and vegetable intake. The reviewed studies demonstrate the potential of nutritional educational interventions to address dietary problems and body image challenges during childhood and adolescence. No distinction due to age was observed, despite taking into account a wide age span.

Such interventions affected food intake behaviors and their related outcomes (RQ1). They improved dietary and nutritional knowledge (17, 20, 21, 24, 25). Empowering adolescents with accurate and relevant nutritional knowledge enables them to make informed dietary choices, resulting in healthier eating patterns. They effectively improved dietary attitudes and practices, leading to increased consumption of nutritious foods and reduced intake of unhealthy options. Many of the nutritional educational interventions also positively influenced physical activity levels among participants. Several studies showed that post-intervention participants had increased physical activity duration and decreased sedentary behavior. This is consistent with previous research which shows that nutritional interventions significantly enhance both physical activity and literacy (27–29). Physical activity is critical in maintaining overall health and preventing obesity and related health issues, making this an important benefit of the interventions. Enhanced literacy skills are crucial in understanding and applying nutritional knowledge, fostering a comprehensive approach to health.

Furthermore, the review found that the interventions significantly reduced obesity and weight gain among Asian adolescents through better food choices, healthier eating habits, and more physical activity. The studies showed a correlation between nutritional education and improved body mass index, waist circumference, and other anthropometric measures (20). Beyond weight loss, the focus is on enhancing overall well-being, recognizing that comprehensive health extends beyond weight management. These anthropometric measures are vital indicators of overall health and well-being. With well-structured nutritional education interventions, adolescents are encouraged to make better dietary decisions, leading to a better long-term lifestyle with less obesity (1, 2) and related disease risk.

The findings also suggested that nutritional education interventions affected body image perception (RQ2). They reduced misperception and increased satisfaction with body image among Asian adolescents (22, 23). These interventions have the potential to address unrealistic ideals and focus on long-term approaches that can reinforce positive mindset changes. Thus, they could be crucial in improving the overall health and well-being of Asian adolescents.

Future policymaking could draw from the findings of this review. In particular, this research underscores the importance of incorporating nutritional education programs in schools, universities, and other educational settings. It demonstrates that interventions can impact adolescents’ eating habits and body image perceptions, setting the foundation for a healthier future if proper education is provided at an early growing phase of development.

Increased awareness, literacy and knowledge about diets, as well as groundwork that empowers and encourages families to get involved, can reinforce the effectiveness of educational interventions. The research showed that creating supportive environments and providing practical tools that enable behavioral changes is crucial, leading to improved overall health and reduced risk of diet-related diseases (4). Well-structured interventions must educate adolescents to understand and interpret nutrition information on food packaging, as well as provide them with a new understanding of the long-term consequences of unhealthy food intake. An effective intervention will also promote behavioral change techniques that help adolescents set goals, plan their meals, and track their progress. Group-based educational interventions can encourage adolescents to adopt healthier eating habits with the support of their peers.

Nutritional knowledge does not always translate into behavioral change, as significant shifts in behavior often demand a multidisciplinary approach. This is consistent with the findings of Yun and colleagues, who demonstrated that although all students from the University Brunei Darussalam knew the importance of a balanced diet, more than 50% of the students skipped breakfast and consumed fried food at least three times a week (30). Hence, the university was encouraged to form a multi-disciplinary team to be responsible for designing and conducting various programs to promote physical activity and reduce unhealthy eating habits.

It is important to note that program design and mode of administration will dictate the effectiveness of any nutritional education intervention. In this sense, continued evaluation of these programs is essential. Interventions must be well targeted in terms of the recipient population and time frame. A regular and repetitive intervention (20) of adequate duration (24) seems to achieve better results. Some studies even suggest that customized nutrition education programs are needed in the future (18). The use of innovative technology specifically adapted to young people such as movies (17), text messaging (18), or mobile apps (19) can be useful from the point of view of participant satisfaction and to increase adherence rates. As such, peer education might be helpful to boost both (24). These innovative solutions are also generally cost-effective, scalable and easy to implement (19). If the intervention is to be successful, it is essential to target feasible objectives and to use clear, simple, and culturally appropriate messages (18, 21). It is also worth mentioning that nutritional interventions aimed at addressing obesity should never conflict with any negative effects on body image (26).

Apart from the large number of interventions and outcomes considered in the present review which demonstrate the potential of nutritional education interventions to address dietary problems and body image, other strengths of the reviewed studies include the fact that some of these innovative solutions have been explored and, overall, have shown positive compliance and a generally high rate of satisfaction, novel data have been collected, and a wide range of different methodological designs supported by robust statistical analysis have been implemented. As a matter of fact, our quality assessment yielded very good results, and all the reviewed articles were rated above 80%. Altogether, this provides a solid basis for future developments.

Notwithstanding the many merits of the methodologies used in the reviewed articles, some limitations emerge. These limitations may affect the generalization of the results, such as the use of relatively small sample sizes (17, 21), homogeneous samples (21), or unbalanced samples across genders (18), or focusing on a specific population (17, 24). They may affect the precision of measurement. For example, self-reported measures may have introduced potential biases (18, 22, 23), it was not possible to assess the quality of education or to ensure that it was provided equally in all cases (22, 24) and one study notes that it may be necessary to keep working on the design of a reliable measure of body image (26). These limitations may also threaten the study’s validity as some experimental designs did not involve random allocation in the intervention and control groups (19, 24, 26). Moreover, potential contamination bias may have occurred (26) and the use of non-blinded methods may have overestimated intervention effects (23). The cross-sectional nature of the study is also acknowledged (19) and some articles agree on the need to conduct future longitudinal designs to assess long-term effects (18).

Further research is needed to validate the benefits of current educational material and to further explore other innovative technologies such as game-based interventions. Furthermore, it would be highly desirable to have more evidence of the suitability and effectiveness of the programs in terms of target population, duration, participation, adherence rates or mode of administration. More empirical analyses of longitudinal data are needed to test the long-run effects of nutrition education interventions in schools, universities and other educational settings and whether they improve eating habits and good health later in life. Finally, further research is needed to investigate the factors that lead to behavior change. While have found that there is consistent evidence of correlations between nutrition knowledge and food intake and body image, we are aware it is also important to consider the different ways in which correlations between nutritional education and food intake or body image may arise.

## 5 Conclusion

This systematic review highlights the impact of nutritional education interventions on improving the overall health and well-being of Asian adolescents by promoting healthier food choices and fostering positive body image perceptions. We strongly recommend continuing efforts to integrate nutritional education programs into educational settings to combat adolescent obesity and related health issues effectively, with a multidisciplinary approach in mind. Further research is needed to investigate the factors that lead to behavior change. While we found that there is consistent evidence of correlations between nutrition knowledge and food intake and body image, we are aware it is also important to consider the different ways in which correlations between nutritional education and food intake or body image may arise. The results of this study emphasize the importance of nutrition education for Asian adolescents to promote healthier choices and maintain a positive body image. Integrating nutrition education into an educational setting is therefore a proactive step to address these issues comprehensively. The far-reaching implications of this research are recommendations for policymakers, educators and healthcare providers. If implemented effectively, nutrition education could significantly improve the health and well-being of Asian adolescents.

## Data availability statement

The original contributions presented in the study are included in the article/supplementary material, further inquiries can be directed to the corresponding author.

## Author contributions

BP: Conceptualization, Data curation, Formal analysis, Investigation, Methodology, Resources, Software, Writing – original draft. SNA: Conceptualization, Formal analysis, Investigation, Methodology, Supervision, Validation, Writing – review & editing. SS: Conceptualization, Methodology, Supervision, Validation, Writing – review & editing. ZM: Supervision, Validation, Writing – review & editing. SRA: Conceptualization, Formal analysis, Investigation, Methodology, Supervision, Validation, Writing – review & editing.

## References

[1] D’Addesa D D’Addezio L Martone D Censi L Scanu A Cairella G . Dietary intake and physical activity of normal weight and overweight/obese adolescents. Int J Pediatr. (2010) 2010:785649. doi: 10.1155/2010/785649, PMID: 20585356 PMC2878668

[2] Pereira HRC Bobbio TG Antonio MRGM Barros Filho ADA. Childhood and adolescent obesity: how many extra calories are responsible for excess of weight? Rev Paul Pediatr. (2013) 31:252–7. doi: 10.1590/s0103-05822013000200018, PMID: 23828064

[3] National Center for Chronic Disease Prevention and Health Promotion (U.S.). Comprehensive framework for addressing the school nutrition environment and services. Atlanta: Centers for Disease Control and Prevention (2019).

[4] Cunha CM Costa PRF de Oliveira LPM Queiroz VAO Pitangueira JCD Oliveira AM. Dietary patterns and cardiometabolic risk factors among adolescents: systematic review and meta-analysis. Br J Nutr. (2018) 119:859–79. doi: 10.1017/s0007114518000533, PMID: 29644953

[5] Vanhelst J Beghin L Duhamel A De Henauw S Ruiz JR Kafatos A . Do adolescents accurately evaluate their diet quality? The HELENA study. Clin Nutr. (2017) 36:1669–73. doi: 10.1016/j.clnu.2016.10.019, PMID: 27842927

[6] World Health Organization. Guideline: sugars intake for adults and children. Geneva: World Health Organization (2015). 25905159

[7] Park JH Hahm M-I Kim SJ Min IS. Association between high-caffeine energy drink intake and suicidal ideation in Korean adolescents. J. Korean Soc. Sch. Health. (2016) 29:71–80. doi: 10.15434/kssh.2016.29.2.71

[8] So ES. Perceptual body image and the relationship with weight control across the adult lifespan by sex in Koreans. J Public Health. (2017) 39:777–86. doi: 10.1093/pubmed/fdx021, PMID: 28334764

[9] Griffiths S Murray SB Bentley C Gratwick-Sarll K Harrison C Mond JM. Sex differences in quality-of-life impairment associated with body dissatisfaction in adolescents. J Adolesc Health. (2017) 61:77–82. doi: 10.1016/j.jadohealth.2017.01.016 28389062

[10] Patton GC Sawyer SM Santelli JS Ross DA Afifi R Allen NB . Our future: a lancet commission on adolescent health and wellbeing. Lancet. (2016) 387:2423–78. doi: 10.1016/S0140-6736(16)00579-1, PMID: 27174304 PMC5832967

[11] Baker JH Higgins Neyland MK Thornton LM Runfola CD Larsson H Lichtenstein P . Body dissatisfaction in adolescent boys. Dev Psychol. (2019) 55:1566–78. doi: 10.1037/dev0000724, PMID: 30985163 PMC6586497

[12] Van den Berg P Paxton SJ Keery H Wall M Guo J Neumark-Sztainer D. Body dissatisfaction and body comparison with media images in males and females. Body Image. (2007) 4:257–68. doi: 10.1016/j.bodyim.2007.04.003 18089272

[13] Bornioli A Lewis-Smith H Smith A Slater A Bray I. Adolescent body dissatisfaction and disordered eating: predictors of later risky health behaviors. Soc Sci Med. (2019) 238:112458. doi: 10.1016/j.socscimed.2019.112458, PMID: 31450163

[14] Haines J Neumark-Sztainer D. Prevention of obesity and eating disorders: a consideration of shared risk factors. Health Educ Res. (2006) 21:770–82. doi: 10.1093/her/cyl094, PMID: 16963727

[15] Page MJ McKenzie JE Bossuyt PM Boutron I Hoffmann TC Mulrow CD . The PRISMA 2020 statement: an updated guideline for reporting systematic reviews. BMJ. (2021) 372:n71. doi: 10.1136/bmj.n71, PMID: 33782057 PMC8005924

[16] Study Quality Assessment Tools (2013). Available at: https://www.nhlbi.nih.gov/health-topics/study-quality-assessment-tools

[17] Marliya M Muhammad HFL. Introducing the new nutrition guideline to Indonesian overweight/obese adolescents using a short movie: the impact on nutritional knowledge, eating habit and dietary intake. Prog Nutr. (2019) 21:227–33. doi: 10.23751/pn.v21i1-S.5944

[18] Shahril MR Wan Dali WPE Lua PL. A 10-week multimodal nutrition education intervention improves dietary intake among university students: cluster-randomised controlled trial. J Nutr Metab. (2013) 2013:658642. doi: 10.1155/2013/658642, PMID: 24069535 PMC3771440

[19] Wang M Guo Y Zhang Y Xie S Yu Z Luo J . Promoting healthy lifestyle in Chinese college students: evaluation of a social media-based intervention applying the RE-AIM framework. Eur J Clin Nutr. (2021) 75:335–44. doi: 10.1038/s41430-020-0643-2, PMID: 32366994

[20] Singhal N Misra A Shah P Gulati S. Effects of controlled school-based multi-component model of nutrition and lifestyle interventions on behavior modification, anthropometry and metabolic risk profile of urban Asian Indian adolescents in North India. Eur J Clin Nutr. (2010) 64:364–73. doi: 10.1038/ejcn.2009.150, PMID: 20087379

[21] Chen JL Guedes CM Lung AE. Smartphone-based healthy weight management intervention for Chinese American adolescents: short-term efficacy and factors associated with decreased weight. J Adolesc Health. (2019) 64:443–9. doi: 10.1016/j.jadohealth.2018.08.022, PMID: 30409751

[22] Lee JH Lee HS Kim H Kwon YJ Shin J Lee JW. Association between nutrition education, dietary habits, and body image misperception in adolescents. Asia Pac J Clin Nutr. (2021) 30:512–21. doi: 10.6133/apjcn.202109_30(3).0018, PMID: 34587711

[23] Koo HC Poh BK Ruzita AT. GReat-child trial™ based on social cognitive theory improved knowledge, attitudes and practices toward whole grains among Malaysian overweight and obese children. BMC Public Health. (2019) 19:1574. doi: 10.1186/s12889-019-7888-5, PMID: 31775696 PMC6881981

[24] Sharif Ishak SIZ Chin YS Taib M Nasir M Chan YM Shariff ZM. Effectiveness of a school-based intervention on knowledge, attitude and practice on healthy lifestyle and body composition in Malaysian adolescents. BMC Paediatr. (2020) 20:122. doi: 10.1186/s12887-020-02023-x, PMID: 32171276 PMC7071695

[25] Tse MMY Yuen DTW. Effects of providing a nutrition education program for teenagers: dietary and physical activity patterns. Nurs Health Sci. (2009) 11:160–5. doi: 10.1111/j.1442-2018.2009.00443.x 19519703

[26] Yeh MC Liou YM Chien LY. Development and effectiveness of a school programme on improving body image among elementary school students in Taiwan. J Adv Nurs. (2012) 68:434–43. doi: 10.1111/j.1365-2648.2011.05735.x, PMID: 21679223

[27] Beets MW Beighle A Erwin HE Huberty JL. After-school program impact on physical activity and fitness: a meta-analysis. Am J Prev Med. (2009) 36:527–37. doi: 10.1016/j.amepre.2009.01.033, PMID: 19362799

[28] Pate RR O'Neill JR. After-school interventions to increase physical activity among youth. Br J Sports Med. (2009) 43:14–8. doi: 10.1136/bjsm.2008.055517 19019903

[29] Egan CA Webster CA Stewart GL Weaver RG Russ LB Brian A . Case study of a health optimizing physical education-based comprehensive school physical activity program. Eval Program Plann. (2019) 72:106–17. doi: 10.1016/j.evalprogplan.2018.10.006, PMID: 30326329

[30] Yun TC Ahmad SR Quee DSQ. Dietary habits and lifestyle practices among university students in Universiti Brunei Darussalam. Malays J Med Sci. (2018) 25:56–66. doi: 10.21315/mjms2018.25.3.6, PMID: 30899187 PMC6422551

